# Supplementary material for: Increased Extracellular Adenosine in Radiotherapy-Resistant Breast Cancer Cells Enhances Tumor Progression through A2AR-Akt-β-Catenin Signaling
Source: Cancers (Basel). 2021 Apr 27;13(9):2105. doi: 10.3390/cancers13092105 (PMC8123845; doi:10.3390/cancers13092105)
Supplement: Supplementary file 1 [file cancers-13-02105-s001.zip › cancers-1189560-supplementary.pdf]

Supplementary Materials

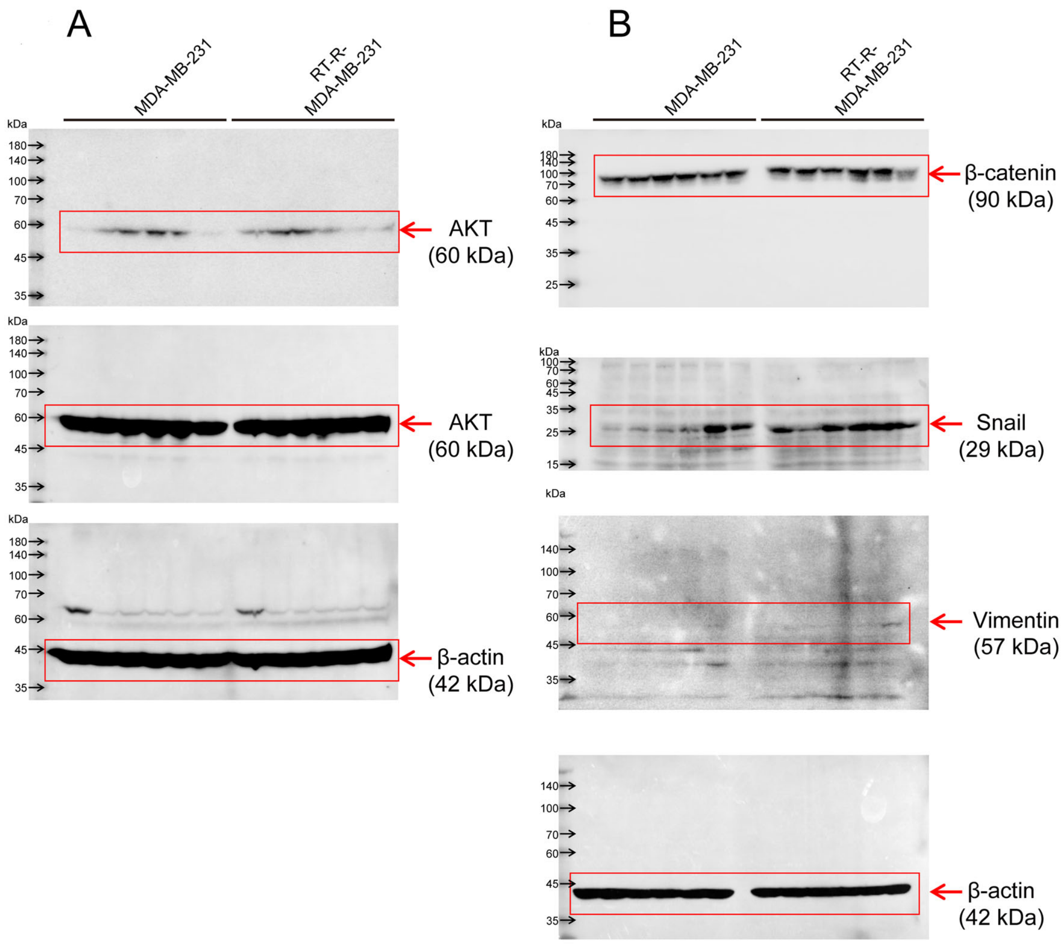

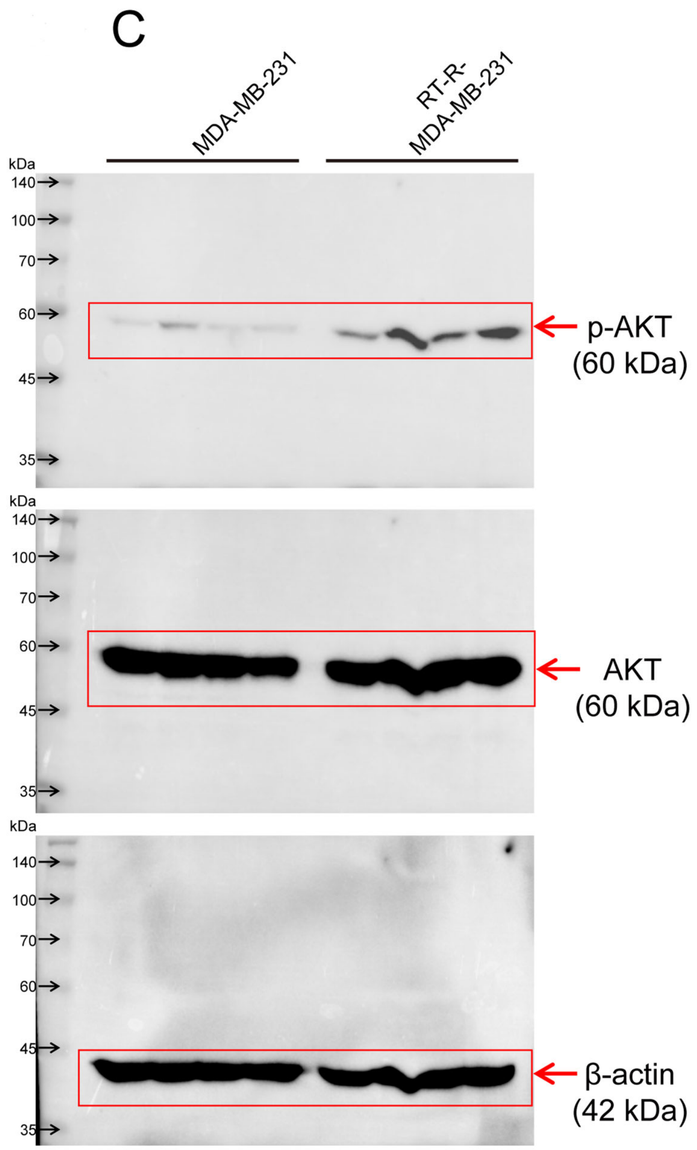
C
MDA-MB-231
RT-R-MDA-MB-231
kDa
140
100
70
60
45
35
p-AKT (60 kDa)
AKT (60 kDa)
β-actin (42 kDa)

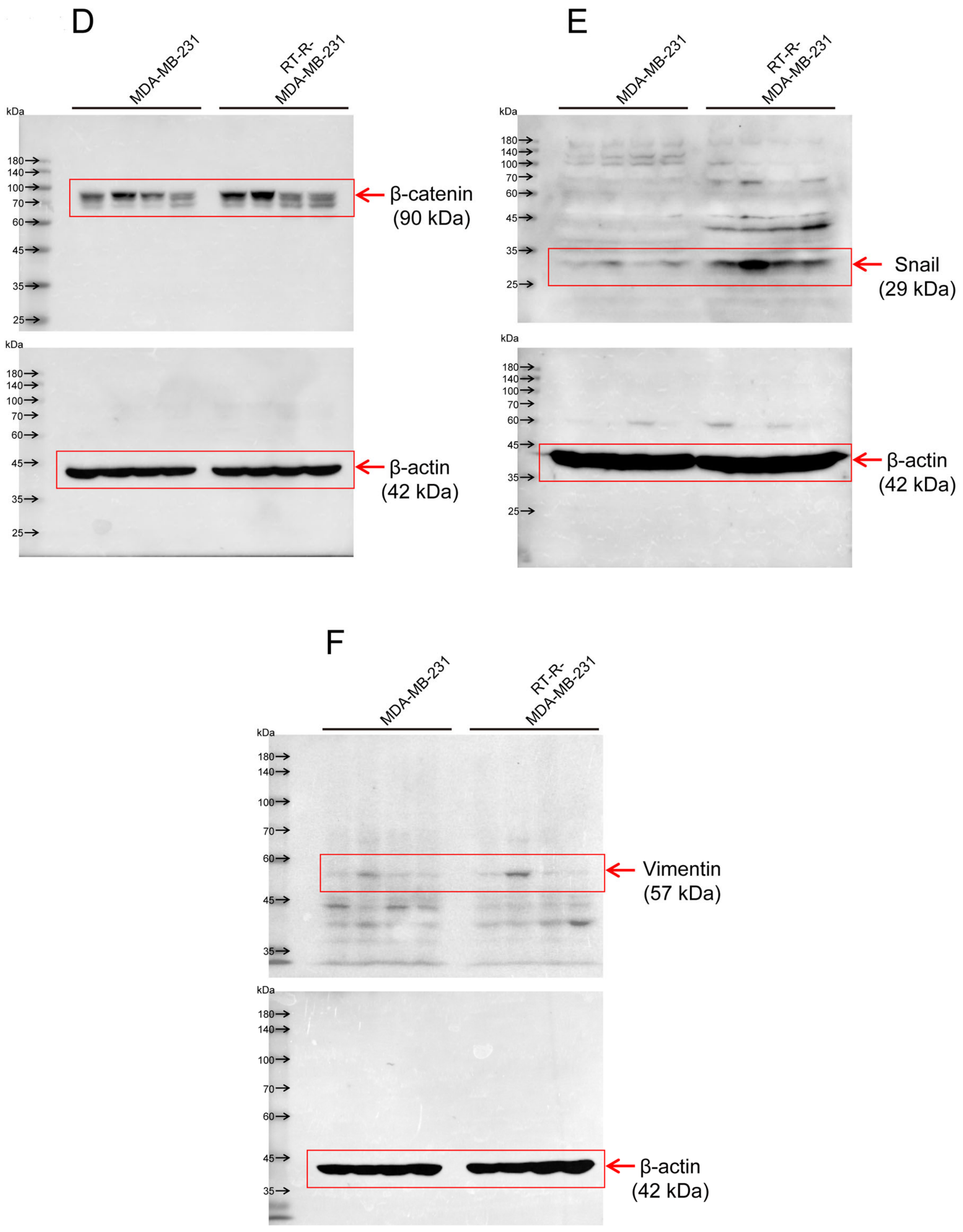

Figure S1. Raw images of Western blotting.

Table S1: Intensity ratios of western blots for Figure 5C

				p-AKT	AKT	p-AKT/AKT	Fold of CTRL
Set 1	MDA-MB-231	CTRL siRNA	-	2347.51	20298.45	0.1156	1
			ADO	5004.72	16935.72	0.2955	2,5552
		A2AR siRNA	-	2357.34	16374.48	0.1440	1,2448
			ADO	2807.41	15824.86	0.1774	1,5340
	RT-R-MDA-MB-231	CTRL siRNA	-	5199.38	18416.98	0.2823	2,4411
			ADO	15224.91	19428.31	0.7836	6,7760
		A2AR siRNA	-	6846.01	19849.72	0.3449	2,9822
			ADO	9478.86	21178.69	0.4476	3,8700
Set 2	MDA-MB-231	CTRL siRNA	-	1514.14	21410.91	0.0707	1
			ADO	3868.74	17349.48	0.2230	3,1532
		A2AR siRNA	-	1491.26	16931.67	0.0881	1,2454
			ADO	2581.26	19278.00	0.1339	1,8934
	RT-R-MDA-MB-231	CTRL siRNA	-	3453.03	12795.40	0.2699	3,8161
			ADO	8336.67	15459.60	0.5393	7,6254
		A2AR siRNA	-	3854.50	16162.38	0.2385	3,3723
			ADO	5600.81	21851.57	0.2563	3,6244
Set 3	MDA-MB-231	CTRL siRNA	-	1394. 01	19304. 08	0.0722	1
			ADO	6059.52	21330.45	0.2841	3.9339
		A2AR siRNA	-	1192.64	15385.26	0.0775	1.0735
			ADO	1161.36	20540.69	0.0565	0.7829
	RT-R-MDA-MB-231	CTRL siRNA	-	5612.83	24500.71	0.2291	3.1724
			ADO	9735.15	20012.60	0.4865	6.7363
		A2AR siRNA	-	5001.55	20559.84	0.2433	3. 3687
			ADO	6687.03	20618.21	0.3243	4. 4912
Set 4	MDA-MB-231	CTRL siRNA	-	1351.44	24424.45	0.0553	1
			ADO	3192.04	22162.50	0.1440	2.6030
		A2AR siRNA	-	1391.85	18448.96	0.0754	1.3635
			ADO	2088.41	18570.40	0.1125	2.0325
	RT-R-MDA-MB-231	CTRL siRNA	-	4336.67	22741.57	0.1907	3.4464
			ADO	8560.03	19660.18	0.4354	7.8690
		A2AR siRNA	-	2945.77	19546.01	0.1507	2.7238
			ADO	4829.74	23458.21	0.2059	3.7210
Set 5	MDA-MB-231	CTRL siRNA	-	1591.55	11205.08	0.1420	1
			ADO	5275.69	13344.33	0.3954	2.7834
		A2AR siRNA	-	1113.23	12786.26	0.0871	0.6130
			ADO	1212.79	13245.05	0.0916	0.6447
	RT-R-MDA-MB-231	CTRL siRNA	-	4784.84	12979.23	0.3687	2.5955
			ADO	10861.86	10081.04	1.0775	7.5857
		A2AR siRNA	-	5851.96	13378.43	0.4374	3.0796
			ADO	7419.38	12282.62	0.6041	4.2528

Table S2: Summary of relative p-Akt/Akt protein levels for Figure 5C

p-AKT/AKT	MDA-MB-231				RT-R-MDA-MB-231			
	CTRL siRNA		A2AR siRNA		CTRL siRNA		A2AR siRNA	
	-	ADO	-	ADO	-	ADO	-	ADO
n= 1	1	2.5552	1.2448	1.5340	2.4411	6.7760	2.9822	3.8700
n = 2	1	3.1532	1.2454	1.8934	3.8161	7.6254	3.3723	3.6244
n = 3	1	3.9339	1.0735	0.7829	3.1724	6.7363	3. 3687	4.4912
n = 4	1	2.6030	1.3635	2.0325	3.4464	7.8690	2.7238	3.7210
n = 5	1	2.7834	0.6130	0.6447	2.5955	7.5857	3.0796	4.2528
**Mean**	**1**	**3.006**	**1.108**	**1.377**	**3.094**	**7.318**	**3,105**	**3.992**
**Std. Deviation**	**0**	**0.5696**	**0.2954**	**0.6345**	**0.5759**	**0.5248**	**0.2748**	**0.3677**

Std. Error of Mean	0	0.2547	0.1321	0.2837	0.2575	0.2347	0.1229	0.1644

Table S3: Intensity ratios of western blots for Figure 5D

				β-catenin	β-actin	β-catenin/β-actin	Fold of CTRL
Set 1	MDA-MB-231	CTRL siRNA	-	5822.43	24244.38	0.2402	1
			ADO	10333.38	20812.84	0.4965	2.0674
		A2AR siRNA	-	4303.26	19334.65	0.2226	0.9268
			ADO	4584.48	19380.98	0.2365	0.9850
	RT-R-MDA-MB-231	CTRL siRNA	-	12804.79	20816.15	0.6151	2.5614
			ADO	19233.55	19682.89	0.9772	4.0689
		A2AR siRNA	-	4559.31	20510.01	0.2223	0.9256
			ADO	5011.72	24831.28	0.2018	0.8404
Set 2	MDA-MB-231	CTRL siRNA	-	6145.67	22149.62	0.2775	1
			ADO	11360.50	20723.67	0.5482	1.9757
		A2AR siRNA	-	6298.79	16937.43	0.3719	1.3403
			ADO	8728.62	19940.45	0.4377	1.5776
	RT-R-MDA-MB-231	CTRL siRNA	-	11032.62	19181.91	0.5752	2.0729
			ADO	17337.67	16995.84	1.0201	3.6766
		A2AR siRNA	-	5247.13	17115.96	0.3066	1.1049
			ADO	5590.74	17103.79	0.3269	1.1781
Set 3	MDA-MB-231	CTRL siRNA	-	5178.50	24911.79	0.2079	1
			ADO	7788.13	19353.01	0.4024	1.9359
		A2AR siRNA	-	5217.01	16283.77	0.3204	1.5412
			ADO	5308.91	18500.57	0.2870	1.3805
	RT-R-MDA-MB-231	CTRL siRNA	-	10901.10	16566.74	0.6580	3.1654
			ADO	14281.38	16977.43	0.8412	4.0467
		A2AR siRNA	-	8513.50	15989.43	0.5324	2.5614
			ADO	8515.08	18129.26	0.4697	2.2595
Set 4	MDA-MB-231	CTRL siRNA	-	6608.08	23308.21	0.2835	1
			ADO	9902.43	20189.67	0.4905	1.7300
		A2AR siRNA	-	7665.08	16658.65	0.4601	1.6230
			ADO	6596.10	16534.50	0.3989	1.4071
	RT-R-MDA-MB-231	CTRL siRNA	-	17259.69	19418.33	0.8888	3.1351
			ADO	22610.67	21903.13	1.0323	3.6412
		A2AR siRNA	-	13705.79	18667.89	0.7342	2.5897
			ADO	14814.91	20999.43	0.7055	2.4884
Set 5	MDA-MB-231	CTRL siRNA	-	4006.55	16249.96	0.2466	1.0000
			ADO	8869.50	18197.96	0.4874	1.9768
		A2AR siRNA	-	3951.08	18318.72	0.2157	0.8748
			ADO	4164.15	17601.45	0.2366	0.9595
	RT-R-MDA-MB-231	CTRL siRNA	-	10369.81	17396.50	0.5961	2.4176
			ADO	17501.91	18696.01	0.9361	3.7968
		A2AR siRNA	-	13364.38	20712.13	0.6452	2.6170
			ADO	14171.79	19199.67	0.7381	2.9937

Table S4. Summary of relative β-catenin/β-actin protein levels for Figure 5D

β-catenin/β-actin	MDA-MB-231				RT-R-MDA-MB-231			
	CTRL siRNA		A2AR siRNA		CTRL siRNA		A2AR siRNA	
	-	ADO	-	ADO	-	ADO	-	ADO
n= 1	1	2.0674	0.9268	0.9850	2.5614	4.0689	0.9256	0.8404
n = 2	1	1.9757	1.3403	1.5776	2.0729	3.6766	1.1049	1.1781
n = 3	1	1.9359	1.5412	1.3805	3.1654	4.0467	2.5614	2.2595
n = 4	1	1.7300	1.6230	1.4071	3.1351	3.6412	2.5897	2.4884

n = 5	1	1.9768	0.8748	0.9595	2.4176	3.7968	2.6170	2.9937
Mean	1	1.937	1.261	1.262	2.671	3.846	1.96	1.952
Std. Deviation	0	0.1254	0.3452	0.2752	0.4727	0.2019	0.8647	0.9086
Std. Error of Mean	0	0.05609	0.1544	0.1231	0.2114	0.09029	0.3867	0.4063

Table S5. Intensity ratios of western blots for Figure 5E

				**Snail**	**β-actin**	**Snail/β-actin**	**Fold of CTRL**
Set 1	MDA-MB-231	CTRL siRNA	-	1048.97	18771.74	0.0559	1
			ADO	2546.03	21940.01	0.1160	2.0767
		A2AR siRNA	-	839.78	25920.26	0.0324	0.5798
			ADO	2007.57	22565.77	0.0890	1.5921
	RT-R-MDA-MB-231	CTRL siRNA	-	5069.10	25855.64	0.1961	3.5085
			ADO	10464.33	17068.53	0.6131	10.9713
		A2AR siRNA	-	6172.72	23217.55	0.2659	4.7577
			ADO	4087.03	26189.50	0.1561	2.7927
Set 2	MDA-MB-231	CTRL siRNA	-	1503.13	20055.28	0.0749	1
			ADO	4796.81	20361.06	0.2356	3.1433
		A2AR siRNA	-	1537.01	20563.57	0.0747	0.9973
			ADO	1665.81	17506.98	0.0952	1.2695
	RT-R-MDA-MB-231	CTRL siRNA	-	4886.81	13975.21	0.3497	4.6655
			ADO	9142.35	11801.77	0.7747	10.3357
		A2AR siRNA	-	5337.84	21116.45	0.2528	3.3727
			ADO	3548.21	26043.15	0.1362	1.8178
Set 3	MDA-MB-231	CTRL siRNA	-	500.09	22525.55	0.0222	1
			ADO	2157.35	23654.43	0.0912	4.1080
		A2AR siRNA	-	508.41	19917.72	0.0255	1.1497
			ADO	505.38	19607.21	0.0258	1.1610
	RT-R-MDA-MB-231	CTRL siRNA	-	818.93	18270.08	0.0448	2.0190
			ADO	4796.08	18540.72	0.2587	11.6516
		A2AR siRNA	-	822.76	17046.77	0.0483	2.1740
			ADO	827.11	18082.84	0.0457	2.0603
Set 4	MDA-MB-231	CTRL siRNA	-	1188.41	21368.38	0.0556	1
			ADO	4768.69	21395.77	0.2229	4.0075
		A2AR siRNA	-	1178.91	20473.26	0.0576	1.0354
			ADO	1534.69	21507.74	0.0714	1.2830
	RT-R-MDA-MB-231	CTRL siRNA	-	5605.62	23243.10	0.2412	4.3365
			ADO	11917.98	17864.11	0.6671	11.9958
		A2AR siRNA	-	3922.13	21335.01	0.1838	3.3055
			ADO	5746.74	20945.57	0.2744	4.9333
Set 5	MDA-MB-231	CTRL siRNA	-	4515.01	22167.81	0.2037	1
			ADO	6635.28	16198.82	0.4096	2.0111
		A2AR siRNA	-	3862.39	22260.33	0.1735	0.8519
			ADO	4102.26	15838.13	0.2590	1.2717
	RT-R-MDA-MB-231	CTRL siRNA	-	12689.43	21119.88	0.6008	2.9499
			ADO	21594.52	15366.99	1.4053	6.8995
		A2AR siRNA	-	10116.13	15259.58	0.6629	3.2549
			ADO	18449.96	22468.03	0.8212	4.0318

Table S6. Summary of relative Snail/β-actin protein levels for Figure 5E

**Snail/β-actin**	**MDA-MB-231**				**RT-R-MDA-MB-231**			
	**CTRL siRNA**		**A2AR siRNA**		**CTRL siRNA**		**A2AR siRNA**	
	**-**	**ADO**	**-**	**ADO**	**-**	**ADO**	**-**	**ADO**
n= 1	1	2.0767	0.5798	1.5921	3.5085	10.9713	4.7577	2.7927
n = 2	1	3.1433	0.9973	1.2695	4.6655	10.3357	3.3727	1.8178

n = 3	1	4.1080	1.1497	1.1610	2.0190	11.6516	2.1740	2.0603
n = 4	1	4.0075	1.0354	1.2830	4.3365	11.9958	3.3055	4.9333
n = 5	1	2.0111	0.8519	1.2717	2.9499	6.8995	3.2549	4.0318
Mean	1	3.069	0.9228	1.315	3.496	10.37	3.373	3.127
Std. Deviation	0	1.009	0.2193	0.1624	1.067	2.043	0.9184	1.327
Std. Error of Mean	0	0.4511	0.09808	0.07261	0.4772	0.9136	0.4107	0.5935

Table S7. Intensity ratios of western blots for Figure 5F

				Vimentin	β-actin	Vimentin/β-actin	Fold of CTRL
Set 1	MDA-MB-231	CTRL siRNA	-	8840.02	18759.28	0.4712	1
			ADO	22037.31	19036.13	1.1577	2,4566
		A2AR siRNA	-	8659.70	17925.99	0.4831	1,0251
			ADO	8142.27	20832.91	0.3908	0,8294
	RT-R-MDA-MB-231	CTRL siRNA	-	9633.89	19870.50	0.4848	1,0289
			ADO	36129.50	16554.75	2.1824	4,6313
		A2AR siRNA	-	8052.17	20666.18	0.3896	0,8268
			ADO	6268.12	21702.98	0.2888	0,6129
Set 2	MDA-MB-231	CTRL siRNA	-	5435.91	16682.21	0.3259	1
			ADO	13803.57	14083.06	0.9802	3.0080
		A2AR siRNA	-	5840.84	16733.60	0.3490	1.0712
			ADO	4451.62	17406.74	0.2557	0.7848
	RT-R-MDA-MB-231	CTRL siRNA	-	5912.04	16216.33	0.3646	1.1188
			ADO	26642.27	15928.94	1.6726	5.1329
		A2AR siRNA	-	6605.14	19916.38	0.3316	1.0178
			ADO	4224.63	21133.69	0.1999	0.6135
Set 3	MDA-MB-231	CTRL siRNA	-	1930.89	23963.15	0.0806	1
			ADO	4267.57	14538.11	0.2935	3.6430
		A2AR siRNA	-	1468.46	19341.96	0.0759	0.9422
			ADO	1015.53	21260.10	0.0478	0.5928
	RT-R-MDA-MB-231	CTRL siRNA	-	1902.18	22278.81	0.0854	1.0596
			ADO	12450.05	21685.43	0.5741	7.1251
		A2AR siRNA	-	1816.18	21011.84	0.0864	1.0727
			ADO	2557.67	16444.65	0.1555	1.9302
Set 4	MDA-MB-231	CTRL siRNA	-	3419.84	16795.26	0.2036	1
			ADO	12471.98	17298.13	0.7210	3.5409
		A2AR siRNA	-	4097.91	17003.68	0.2410	1.1836
			ADO	3181.84	18003.68	0.1767	0.8680
	RT-R-MDA-MB-231	CTRL siRNA	-	4285.48	14683.74	0.2919	1.4333
			ADO	21219.71	15557.23	1.3640	6.6987
		A2AR siRNA	-	5051.01	19533.41	0.2586	1.2699
			ADO	4198.48	18702.94	0.2245	1.1025
Set 5	MDA-MB-231	CTRL siRNA	-	4812.55	16657.26	0.2889	1.0000
			ADO	12682.86	14787.48	0.8577	2.9686
		A2AR siRNA	-	5397.79	17081.60	0.3160	1.0937
			ADO	4861.03	18191.15	0.2672	0.9249
	RT-R-MDA-MB-231	CTRL siRNA	-	5481.71	17103.45	0.3205	1.1093
			ADO	22022.52	16087.94	1.3689	4.7380
		A2AR siRNA	-	5534.67	20805.67	0.2660	0.9207
			ADO	6832.84	21093.03	0.3239	1.1212

Table S8 . Summary of relative Vimentin/β-actin protein levels for Figure 5F

Vimentin/β-actin	MDA-MB-231				RT-R-MDA-MB-231			
	CTRL siRNA		A2AR siRNA		CTRL siRNA		A2AR siRNA	
	-	ADO	-	ADO	-	ADO	-	ADO
n= 1	1	2.4566	1.0251	0.8294	1.0289	4.6313	0.8268	0.6129
n = 2	1	3.0080	1.0712	0.7848	1.1188	5.1329	1.0178	0.6135
n = 3	1	3.6430	0.9422	0.5928	1.0596	7.1251	1.0727	1.9302
n = 4	1	3.5409	1.1836	0.8680	1.4333	6.6987	1.2699	1.1025
n = 5	1	2.9686	1.0937	0.9249	1.1093	4.7380	0.9207	1.1212
Mean	1	3.123	1.063	0.8	1.15	5.665	1.022	1.076
Std. Deviation	0	0.4812	0.08885	0.1267	0.1626	1.163	0.1676	0.5387
Std. Error of Mean	0	0.2152	0.03974	0.05668	0.07271	0.5202	0.07497	0.2409
